# The Impact of O6-Methylguanine-DNA Methyltransferase (*MGMT*) Promoter Methylation on the Outcomes of Patients with Leiomyosarcoma Treated with Dacarbazine

**DOI:** 10.3390/cells12121635

**Published:** 2023-06-15

**Authors:** Lucia Cannella, Rosa Della Monica, Antonella Lucia Marretta, Domenico Iervolino, Bruno Vincenzi, Anna Rosaria De Chiara, Ottavia Clemente, Michela Buonaiuto, Maria Luisa Barretta, Annabella Di Mauro, Massimiliano Di Marzo, Michele Guida, Giuseppe Badalamenti, Lorenzo Chiariotti, Salvatore Tafuto

**Affiliations:** 1 S.C. Sarcomi e Tumori Rari, Istituto Nazionale Tumori—IRCCS—Fondazione “G. Pascale”, 80131 Naples, Italy; l.cannella@istitutotumori.na.it (L.C.); ottavia.clemente@istitutotumori.na.it (O.C.); 2 CEINGE—Biotecnologie Avanzate, 80131 Naples, Italy; dellamonica@ceinge.unina.it (R.D.M.); buonaiutom@ceinge.unina.it (M.B.); lorenzo.chiariotti@unina.it (L.C.); 3 Department of Clinical and Surgery Oncology Unit, University of Naples “Federico II”, 80131 Naples, Italy; antomarretta@live.com; 4 S.C. Anatomia Patologica, IsIstituto Nazionale Tumori—IRCCS—Fondazione “G. Pascale”, 80131 Naples, Italy; domenico.iervolino608@gmail.com (D.I.); annabella.dimauro@istitutotumori.na.it (A.D.M.); 5 Dipartimento di Oncologia Medica, Campus Bio-Medico University, 00168 Rome, Italy; b.vincenzi@policlinicocampus.it; 6 S.S.D Istopatologia dei Linfomi e dei Sarcomi, Istituto Nazionale Tumori—IRCCS—Fondazione “G. Pascale”, 80131 Naples, Italy; a.dechiara@istitutotumori.na.it; 7 S.C. Radiologia, Istituto Nazionale Tumori—IRCCS—Fondazione “G. Pascale”, 80131 Naples, Italy; m.barretta@istitutotumori.na.it; 8 S.C. Chirurgia Addominale, Istituto Nazionale Tumori—IRCCS—Fondazione “G. Pascale”, 80131 Naples, Italy; m.dimarzo@istitutotumori.na.it; 9 Unità Tumori Rari e Melanoma, IRCCS Istituto Tumori “Giovanni Paolo II”, Viale O. Flacco, 65, 70124 Bari, Italy; m.guida@oncologico.bari.it; 10 Department of Surgical, Oncological and Oral Sciences, Section of Medical Oncology, University of Palermo, 90129 Palermo, Italy; giuseppe.badalamenti@unipa.it

**Keywords:** leiomyosarcoma, soft-tissue tumor, dacarbazine, O6-methylguanine-DNA methyltransferase, *MGMT*

## Abstract

Dacarbazine is an important drug in the therapeutic landscape of leiomyosarcoma (LMS). Alkylating agents are subjected to resistance mechanisms based on anti-apoptotic pathways and repair mechanisms, including the DNA repair enzyme O6-methylguanine-DNA methyltransferase (*MGMT*). In this retrospective study, the methylation status of the *MGMT* promoter in histological tumor samples from patients with LMS, dacarbazine-based regimens-treated, was measured and correlated with clinical outcomes aimed at optimizing the use of dacarbazine in soft tissue sarcomas. The patients with unmethylated *MGMT* had better outcomes than those with methylated *MGMT*. Patients without *MGMT* methylation had better Progression Free Survival (PFS) when aged ≥62 years compared to those aged <62 years, while PFS of patients with methylated *MGMT* was less favorable independently of age (*p* = 0.0054). The patients without a methylated *MGMT* gene had higher Disease control rate (DCR). These results are not in agreement with the role of the methylated *MGMT* gene in other tumors, and with this study, we demonstrated the correlation between methylated *MGMT* and poor prognosis; despite that, sample smallness, heterogeneity of LMS and of treatment history could be selection bias. Predictive markers of response to chemotherapies in sarcomas remain an unmet need.

## 1. Introduction

Soft-tissue sarcomas are a heterogeneous group of malignant tumors, including more than 70 different histotypes, with specific biological and clinical behavior. Leiomyosarcoma (LMS) represents 10–20% of all tumors in this group and has an annual incidence of approximately 0.7 per 100,000 [1]. LMS arises from smooth muscle or its precursors and can develop anywhere in the body. The most frequent onset sites are the uterus, retroperitoneum and extremities [2]. Therefore, usually, LMS can be classified into “extrauterine” (retroperitoneal, gastrointestinal, extremity, or subcutaneous) and “uterine” LMS, each with distinct clinicopathological features [3].

Although in most studies on leiomyosarcoma the molecular characteristics of the various sites of onset in correlation with the response to treatment have not been explicitly mentioned, we can affirm that the uterine site has a better sensitivity to chemotherapy treatment also due to the diversity of grading and aggressiveness to diagnosis and that sites such as the LMS of the wall of the vena cava are instead very resistant to all chemotherapy treatments.

Localized disease treatment is based on surgery, possibly with chemotherapy, but relapse is frequent, and the prognosis for patients with advanced disease is poor, with a median overall survival of approximately 19 months [4].

The diagnosis and staging of patients with LMS are in line with general recommendations for STS and visceral sarcomas, and the overall management of LMS patients should be part of a multidisciplinary team in a referral center for high-volume sarcoma.

In general, LMS has not yet been identified a specific target, and this can be one of the handicaps in effective targeted drugs’ developments.

Patients with metastatic disease are treated with chemotherapy, and first-line treatment is based on anthracycline alone or in combination with ifosfamide or dacarbazine, according to current guidelines [5,6]. Several authors observed that dacarbazine exerts a good antitumor activity alone or in combination with gemcitabine [7,8,9]. Although definite evidence is lacking, dacarbazine is often used in combination with doxorubicin as a first-line treatment for advanced LMS, based on an old trial where the combination achieved a response rate of 30% (six of 20 evaluable patients) [5,6,10,11]. Later phase III clinical trials have also confirmed the efficacy of dacarbazine in the LMS subgroup of soft tissue sarcomas [7,8].

A family of targeted agents that has been widely evaluated have been multi-target tyrosine kinase inhibitors, perhaps primarily VEGFR inhibitors. However, few have achieved encouraging results as monotherapy. The multi-targeted tyrosine kinase inhibitors are a family of targeted agents that has been widely assessed; Pazopanib and Regorafenib are two such drugs, but usually they are administered as third or fourth line of therapy. An innovative strategy could be to combine the target therapy with chemotherapy. Although the rationale for combination therapies in sarcomas has strong appeal, unfortunately, the activity of combinations in this setting has not always been demonstrated, and the toxicity profile may be unacceptable.

Therefore, despite other new registered drugs, such as eribulin and trabectedin, dacarbazine remains one of the most important drugs in the therapeutic landscape of sarcomas [12,13].

The recent retrospective study of the European Organization for Research and Treat-ment of Cancer Soft Tissue and Bone Sarcoma Group (EORTC-STBSG), first-line in advanced and metastatic leiomyosarcoma, compared various chemotherapy regimens, including doxorubicin plus dacarbazine and doxorubicin plus ifosfamide, and doxorubicin alone confirmed the superiority in terms of ORR and PFS of dacarbazine with doxorubicin although with the limitations of a multicenter retrospective study.

For this reason, to give value to the combination of doxorubicin with dacarbazine, we analyzed the mechanism of action of alkylating agents and the epigenetic mechanisms that regulate the expression of resistance genes to these agents to identify in the different types of LMS if there is a different expression of these genes and therefore a different response to treatments.

Alkylating agents, such as dacarbazine and its derivative temozolomide, are subjected to resistance mechanisms due to the activation of general anti-apoptotic pathways and repair mechanisms, including the DNA repair enzyme O6-methylguanine-DNA methyltransferase (*MGMT*) [14]. *MGMT* gene is located on chromosome 10q26.3.23-25, and its expression is mainly regulated by epigenetic mechanisms.

The loss of MGMT expression is, only in rare cases, due to gene deletion, mutation or rearrangement; mostly is due to methylation of the CpG island, and several studies demonstrate that CpG island is located in the *MGMT* promoter. Promoter methylation of *MGMT* is associated with the repression of gene transcription and an absence of enzyme production. Loss of enzymatic activity of *MGMT* is correlated with prolonged survival of patients affected by glioblastoma that received temozolomide treatment; in fact this mechanism is a valid help to induce, by using temozolomide, DNA damage in glioblastoma with consequent cellular death. Contrariwise, the function of temozolomide is nullified by MGMT expression: The correct sequence of DNA is restabilized by removing the alkyl groups from the O6 position of guanine [15,16,17]. Hegi et al. found that glioblastoma patients with methylated *MGMT* promoters benefited from temozolomide. In contrast, those who did not have a methylated *MGMT* promoter did not have such a benefit [15]. Currently, the methylation status of the *MGMT* promoter or *MGMT* activity is used as a prognostic predictor factor for the outcome of glioblastoma patients undergoing chemotherapy based on temozolomide [18,19]. There is a lack of data on the importance of *MGMT* methylation in LMS, with the exception of very few dated experiences on a very small series of patients [20].

It is common practice in multimetastatic patients that, upon reaching the maximum dose of anthracycline, achieving a partial response or stability of disease (assessed according to Recist 1.1 criteria), chemotherapy treatment with dacarbazine alone is continued until progression or unacceptable toxicity.

In preclinical studies in glioblastoma, the long-term use of temozolomide (>20 months) showed potentiation of cytotoxic activity by cumulative reduction of the cell’s ability to repair DNA damage [15,16,17].

The present study, which also includes patients treated with “maintenance” dacarbazine, also allows us to investigate whether there is a reduction in the metastasizing potential of dacarbazine in patients treated even after the end of treatment with the anthraciline + dacarbazine combination.

Aiming at optimizing the use of dacarbazine in soft tissue sarcomas and to find out if the methylation of *MGMT* correlates with clinical response, the assessment of *MGMT* promoter methylation may be explored as a possible predictive factor for the identification of patients who may better benefit from chemotherapy. With this objective, methylation status of the *MGMT* promoter in histological tumor samples obtained from patients with LMS, dacarbazine-based regimens treated were measured and correlated with clinical outcomes.

## 2. Patients and Methods

This was a retrospective, multicenter study on metastatic LMS. It was carried out at the National Cancer Institute of Naples—Fondazione G. Pascale, Naples, Italy; the Campus Biomedico of Rome, Roma, Italy; the Oncology Institute of Bari, Bari, Italy; and the University of Palermo, Palermo, Italy. The patient recruitment started in January 2021 and was concluded in January 2022.

The study was conducted in line with the updated Declaration of Helsinki (2013) and the Guidelines for Good Clinical Practice CPM/ICH135/95-DM 15/7/97, in accordance with Legislative Decree no. 200 of 6 November 2007, Implementation of Directive 2005/2/EC Article 3 and GDPR EU Regulation no. 2016/679. The confidentiality of personal and clinical data was guaranteed, in compliance with the EU privacy legislation, and patients released their informed consent to inclusion in the study and publication of anonymous data. The National Cancer Institute of Naples Ethics Committee was notified of the study on 13 January 2021 (D.D. N. 53/2021_C.T.gov number: NCT04893356).

Patients with metastatic histologically diagnosed LMS were recruited between 2010 and 2020 and treated with dacarbazine alone or with anthracyclines, with available tumor tissue samples.

### 2.1. Assessment

The tumor response to treatment was evaluated by the RECIST 1.1 criteria as complete response (CR), partial response (PR), stable disease (SD) or progressive disease (PD). The following parameters were recorded: progression-free survival (PFS; defined as the time from the administration of the first dose of dacarbazine-based regimen to documented radiological progression, death or lost to follow-up, whichever occurred first), overall survival (OS; defined as the time elapsed between the date of diagnosis of the disease and the date of death from all causes or lost-to-follow-up, whichever occurred first) and disease control rate (DCR; defined as the sum of CR, PR and SD > 6 months).

### 2.2. Tumor Tissue Samples Analyses

Tumor tissue samples were fixed in 10% formalin and included in paraffin blocks. Human DNA was extracted from tumor tissues using the FFPE DNA Tissue Kit (Qiagen, Hilden, Germany), following the manufacturer’s instructions. According to the manufacturer’s instructions, genomic DNA (500 ng) was converted with the EZ DNA Methylation Gold Kit (Zymo Research, Irvine, CA, USA). Methylation analysis of tissue samples was performed by performing a Methylation Specific PCR (MSP analysis). To perform the *MGMT* methylation test, we used a nested PCR, as described by Esteller et al. [21]. Briefly, after bisulfite conversion, DNA was amplified using a specific set of primers (forward 5′-GGATATGTTGGGATATAGTT-3′ and reverse primer 5′-CCATCCACAATCACTACAAC-3′). A PCR sample mix, without DNA, was used as reaction negative control. After the reaction, the products of the first PCR were used as templates for the next PCRs. In particular, two PCR reactions were performed, the first was able to recognize methylated CpGs, and the other was able to recognize unmethylated CpGs, using specific pairs of primers (METH-primers: forward primer 5′-GCACTCTTCCGAAAACGAAACG-3′ reverse 5′-GCACTCTTCCGAAAACGAAACG-3′, UNMETH-primers: forward 5′-TTTGTGTTTTGATGTTTGTAGGTTTTTGT-3′ and reverse 5′-ACTCCACACTCTTCCAAAAACAAAACA-3′). PCR products were analyzed with specific controls, fully methylated and unmethylated DNA. PCR products were loaded directly onto 3% agarose gels, stained with ethidium bromide (Sigma-Aldrich, St. Louis, MO, USA) and examined under ultraviolet illumination (Bio-Rad, Hercules, CA, USA). The presence of signal in the lane in which we loaded PCR products of METH primers containing mixes was evaluated to assess the presence of MGMT methylation. The limitation of this technique is that it is a qualitative technique, allowing only to evaluate the presence or absence of MGMT promoter methylation. However MSP is considered the gold standard technique used for MGMT promoter methylation detection in glioblastomas because, although a simple and inexpensive method to assess methylation, it has been demonstrated that the sensitivity and reproducibility is comparable to other methods [22].

### 2.3. Immunohistochemistry (IHC) for MGMT Protein

IHC was performed on 4 µm sections of FFPE tumor blocks. Slides were then deparaffinized in xylene and rehydrated through graded alcohols. Antigen retrieval was performed in Epitope Retrieval Solution pH 9 (×10 concentration—Dako system) at 110 °C for 10 min in TBS and endogenous peroxidase was inactivated with 3% hydrogen peroxide. Slides were incubated with mouse monoclonal *MGMT* primary antibody (MT 3.1, Invitrogen, Waltham, MA, USA) used at a final dilution of 1:250 for 1 h after protein blocking (BSA 5% in PBS 1×). Diaminobenzidine, as a chromogenic substrate, was used to visualize immunoreactivity. Finally, the sections were lightly counterstained with hematoxylin and mounted. One pathologist, blinded to methylation pattern and other parameters, evaluated and scored MGMT expression in the tumor sections. Staining for MGMT protein was considered to be positive if the MGMT staining was evenly distributed in the cell nuclei. Negative staining was defined as staining restricted to the cytoplasm and granular nuclear reactivity.

### 2.4. Statistical Methods

The analyses were carried out using the software R version 4.1.1 (10 August 2021). An alpha of 5% was considered for all associations. Given the small number of samples, comparisons between groups were carried out using non-parametric or semiparametric methods.

Numerical variables were described by the median and interquartile range (IQR); the Wilcox test was used to detect differences between groups. Qualitative variables were described through absolute and relative frequencies, and the comparison between groups was carried out using the exact chi-square test or the exact fisher test when appropriate.

Survival was evaluated by the Kaplan-Meier method, and the log-rank-test performed the comparison between curves. HR estimation was performed using the univariate cox model. The assumption of proportional hazards was verified graphically and through the Harrel and Lee test.

An exploratory analysis was conducted for the variables age, sex, tumor type and radiotherapy to test for the presence of effect modification with *MGMT* methylation.

## 3. Results

Overall, 32 patients with LMS diagnoses were included. Demographic characteristics are reported in Table 1. Thirteen (34.4%) subjects were males, and the median age was 58 years (IQR, 48.75–67.50 years). Our patients were affected by: uterine LMS (n°5),retroperitoneal LMS (n°5), pelvic LMS (n°5), inferior extremities LMS (n°3), other sites (n°6) and not specified LMS (n°8).

Nineteen (53.1%) patients had received radiotherapy: 1 (3.3%) patient in an adjuvant setting, 7 (21.9%) patients in the metastatic setting and 11 (34.4%) patients received palliative radiotherapy in unspecified settings. Dacarbazine was administered in the first line in 17 (53.1%) patients and in further lines in the remaining 15 subjects; median cycles administered were 5 (2–10). Dacarbazine was used only as a monotherapy (Dacarbazine 450 mg/mq day 1–2 every 21 days) in 5 (15.6%) patients and, at the same dose, in combination with anthracycline in 25 (78.1%) patients; more specifically, 11 patients were treated with epirubicin 45 mg/mq day 1–2 every 21 days; and 14 patients with doxorubicin 75 mg/mq day 1 every 21 days. Moreover, dacarbazine was administered in 3 (9.4%) patients both in combination with anthracycline and as monotherapy due to the achievement of anthracycline’s maximum tolerated dose for a period of about 10 months. Twenty-eight (87.5%) patients received surgical treatment.

The best response to treatment was a partial response (PR) in 5 (12.5%) patients and stable disease (SD) in 20 (62.5%) patients, with 78.1% of disease control rate (DCR); 7 (18.8%) patients had a disease progression (PD).

Methylation of the *MGMT* gene was demonstrated in tissue samples from 12 (37.5%) patients. Patients carrying the methylation status had a higher median age than those without methylation, without showing statistical significance (68.5 vs. 56.5 years, *p* = 0.220). No other demographic or clinical variable was significantly correlated in the two groups of patients according to the methylation status of the *MGMT* promoter. Nevertheless, DCR was obtained in 17 (85.0%) patients with un-methylated *MGMT* and 8 (66.7%) of those with methylated *MGMT* promoters (Table 2).

**Figure 1:**
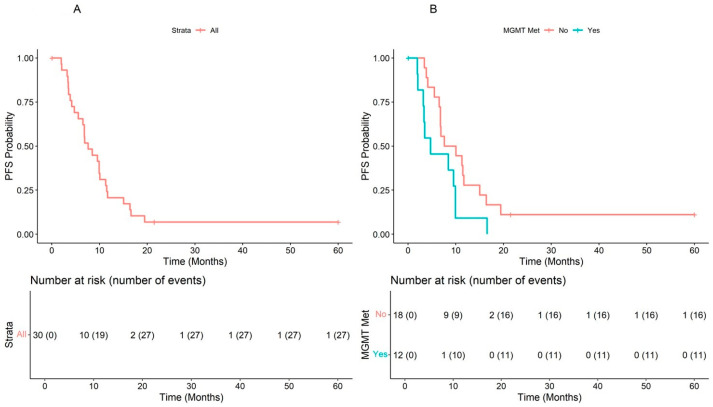
Progression-free survival in the overall population (**A**) and according to *MGMT* methylation status (**B**).

**Figure 2:**
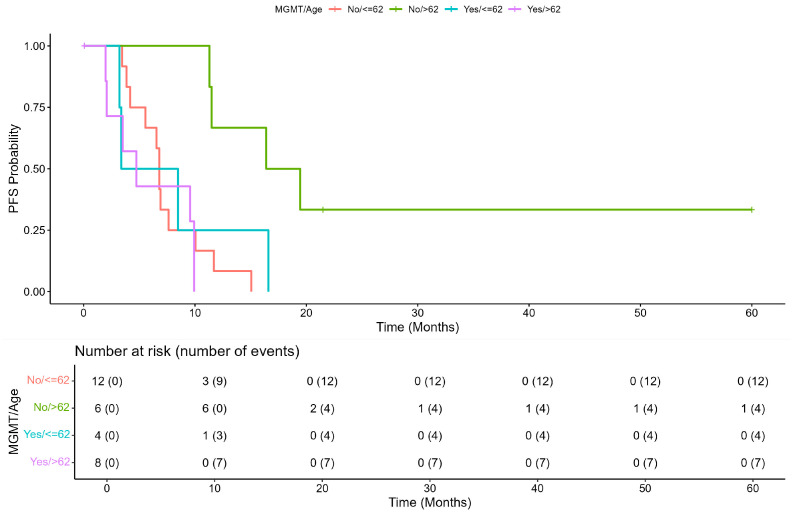
Progression-free survival according to age and to *MGMT* methylation status.

**Figure 3:**
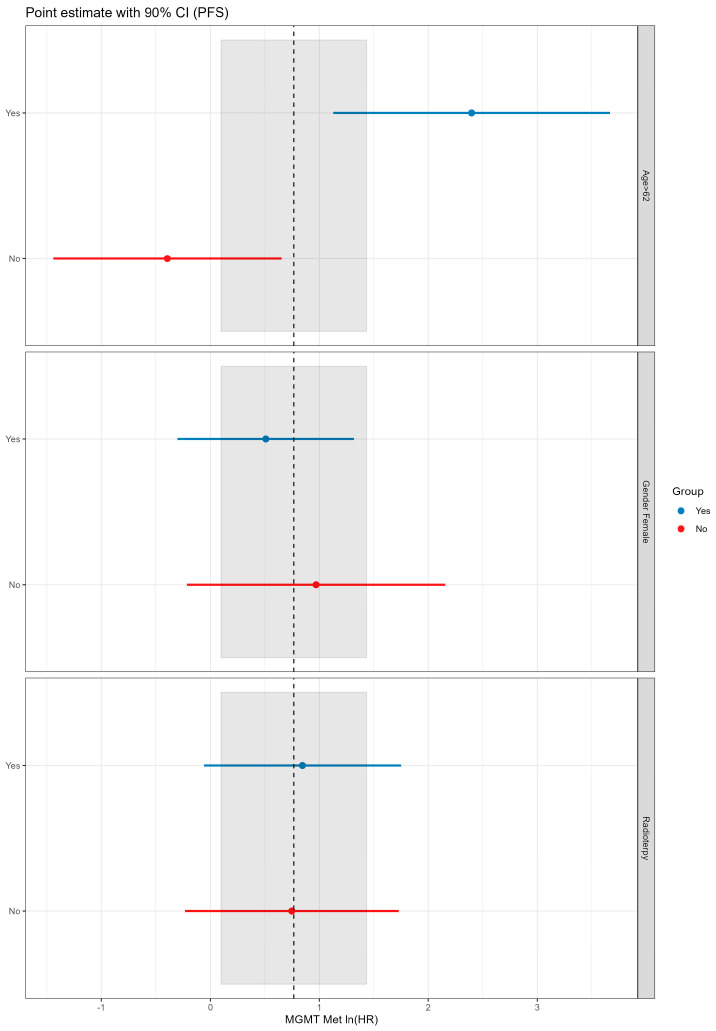
Exploratory subgroup analysis for the effect of methylation on progression-free survival.

The median PFS of the overall population was 7.62 (range, 6.54–11.3) months. The Kaplan Meier plot of PFS showed a difference close to significance between patients with unmethylated *MGMT*, median PFS = 8.84 (95% CI: 6.80–16.4) and patients with methylated *MGMT*, median = 4.73 (95% CI: 3.38–NA), (2.2 (1.00–4.8), *p* = 0.052). The Schoenfeld test confirmed that the risk was independent of time (Figure 1A,B). An exploratory analysis showed that an age ≥62 years was an effect modifier for the methylation status of *MGMT* (*p* = 0.009 vs. age < 62 years), and patients with age >62 years and without methylation of *MGMT* seemed to have a better prognosis (Figure 2 and Figure 3).

Furthermore, the Kaplan-Meier plot showed that patients without MGMT methylation had better PFS when aged ≥62 years compared to those aged <62 years. PFS of patients with methylated *MGMT* was less favorable independently of age (*p* = 0.0054; Figure 2).

**Figure 4:**
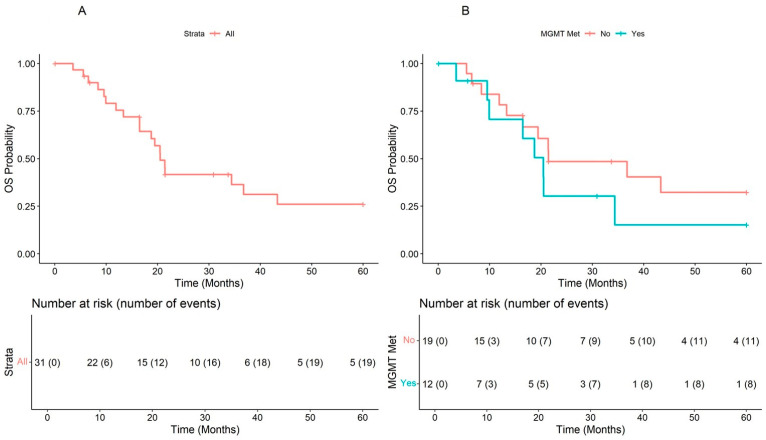
Overall survival in the overall population (**A**) and according to *MGMT* methylation status (**B**).

In the overall population, the median OS was 20.5 (range, 16.5–NA) months, and the patients with unmethylated *MGMT* had better survival at the Kaplan-Meier plot (21.5 months CI: 16.49–NA), but the difference was not significant (HR = 1.7, 95% CI: 0.66–4.2, *p* = 0.28), independently from time (Figure 4A,B).

### Correlation of MGMT Methylation and Its Protein Expression

**Figure 5:**
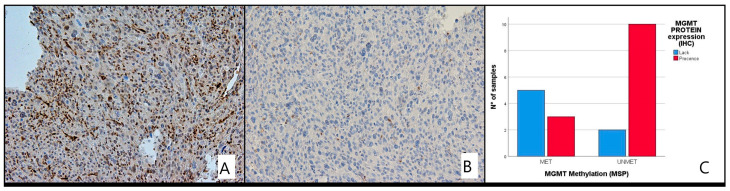
MGMT protein expression by immunohistochemistry (IHC). (**A**) MGMT showing full expression of the protein in tumor cells with unmethylated. (**B**) The majority of tumor cells lack MGMT protein expression with its methylated MGMT promoter. (**C**) Table of contiguity between methylation patterns and MGMT protein distribution.

**Figure 6:**
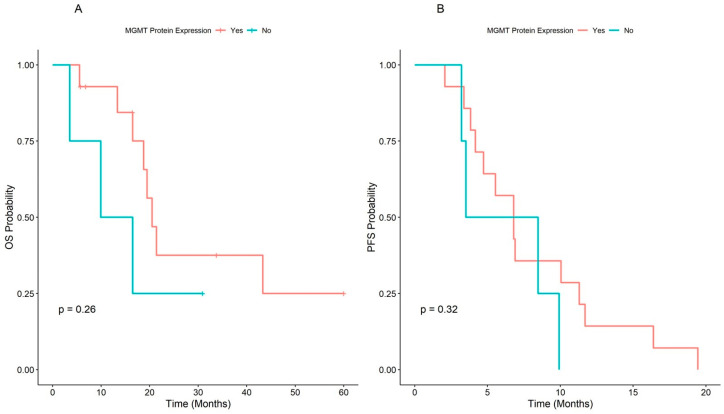
Kaplan Meier curves for Overall Survival (**A**) and Progression Free Survival (**B**) stratified by MGMT protein expression.

MGMT protein expression by IHC was only performed for 20/32 LMS patients due to unavailability of material. We compared the immunohistochemical results with those of the methylation analysis and assumed that the concordant findings between IHC and MS were the absence of MGMT protein expression in the presence of MGMT methylation (MET) and the presence of expression in the absence of MGMT methylation (UNMET) (Figure 5 and Figure 6A,B).

Of the 20 patients evaluated with IHC, 12 were unmethylated and 7 methylated. Loss of protein expression was found in 7 cases (28.6%), while 13 (71.4%) patients had intact MGMT with complete expression. There was a good correlation between the pattern of methylation and protein expression status (*p* < 0.035). No significant differences in protein expression levels were detected in different age groups, gender or between any histological type of LMS (Table 3).

## 4. Discussion

Here, we have studied the methylation status of the *MGMT* promoter in histological tumor samples obtained from patients with LMS treated with dacarbazine, aiming to ameliorate dacarbazine use in these STS and to find out if the methylation of *MGMT* correlates with clinical response. We hypothesized that the assessment of *MGMT* promoter methylation could be explored as a possible predictor for identifying patients most likely to benefit from chemotherapy.

Contrary to the study hypothesis, this retrospective study on 32 patients with LMS treated with dacarbazine found that patients with unmethylated *MGMT* had better outcomes than those with methylated *MGMT*, even if these differences are not significant from a statistical point of view. Indeed, DCR was more frequently attained in patients not carrying a methylated *MGMT* gene promoter than those with a methylated *MGMT*. Additionally, regardless of histotype, we demonstrated that the patients with methylated *MGMT* had a similarly poor PFS, independently of age, while patients without methylation had a better outcome, especially when older than 62 years. Moreover, the difference in mOS between patients with and without methylation of MGMT had a trend in agreement with the effect of unmethylated *MGMT* on mPFS although it was not significant. It should always be considered that heterogeneity of LMS, sample smallness and of treatment history could be selection bias.

In general, MGMT is a small protein present not only in the nucleus but also in the cytoplasm, it repairs O6-alkylguanine adducts independently of any other protein or cofactors; thanks to its mechanism of action, MGMT is also able to protect cancer cells from chemotherapeutic alkylating agents. The expression of MGMT in tissues is variable, for example, there is a high protein expression in liver and lower expression in hematopoietic tissues; therefore, tumor MGMT expression is immensely variable, and consequently, its main role in treatment with alkylant agents.

Although IHC has not been validated in glioblastoma due to a number of limitations, we performed an exploratory evaluation of MGMT protein expression by IHC where possible. A significant correlation between MGMT hypermethylation and MGMT protein expression was identified by IHC (*p* < 0.035).

The different relationship of *MGMT* methylation with the efficacy of alkylating agents on glioblastoma and LMS could be related to the importance of molecular mechanisms in the two settings. An antiangiogenetic activity may be preponderant in glioblastoma, while a cytotoxic effect is more relevant in LMS; *MGMT* methylation could overcome the repair mechanism role in glioblastoma, resulting in an impairment of angiogenesis, and would not be able to counteract tumor cell proliferation [23,24].

Also Mismatch Repair (MMR) status could influence the response of cells to alkylant agents; MMR is the recognition and correction of mispaired bases and deletion / insertion loops generated during DNA synthesis. MMR is of clinical significance in several cancers (including colorectal, ovarian and gastric cancers), LMS and in general STS, could be differently involved in these mechanisms.

Another possibility is that, in LMS, the *MGMT* activity may be substituted by other DNA repair mechanisms that would confer to these tumors dacarbazine resistance even in the presence of *MGMT* methylation [25]. In fact, it has been reported that another gene, ROCK2, often overexpressed in some types of sarcomas, may act as a DNA repair gene when *MGMT* is repressed, providing dacarbazine resistance to sarcomas [25,26].

In addition, the evaluation of *MGMT* promoter methylation in this setting is potentially limited by selection bias because of the smallness and heterogeneity of LMS (uterine vs. extrauterine) and the heterogeneous treatment history of the patients in the study.

Conflicting, or at least controversial, data about the importance of *MGMT* methylation, compared to what happens in glioblastomas, are also in other oncological settings, such as in pancreatic neuroendocrine neoplasms, strongly related to their heterogeneity [17].

## 5. Conclusions

The prognostic and outcome-predictive role of the methylated MGMT gene has been demonstrated in many neoplastic histological types. Unlike expected, the data obtained in this study, although not statistically significant, demonstrate that DCR, median PFS and median OS perform better in LMS patients with unmethylated MGMT compared to methylated MGMT status.

Usually, conflicting data on the role of MGMT methylation, with respect to what occurs in glioblastomas, are also present in other oncological contexts, especially when analyzing very heterogeneous histotypes.

Of course, the retrospective study design, the small sample size, the heterogeneity of LMS and treatment history could be selection biases. However, this is the first study investigating the role of MGMT methylation in LMS as a possible predictive factor for identifying patients who are likely to benefit most from dacarbazine-based regimens.

The research and definition of predictive biomarkers are still unmet needs, therefore, especially in these rare pathologies, further efforts are needed to have more data available not only to continue the research for the identification of tumor markers but also to concretely help the clinicians in the treatment of rare diseases.

## Figures and Tables

**Table 1:** Demographic and clinical characteristics in the overall population.

Characteristics	n = 32, n (%)
Age (years), median (IQR)	58.00 (48.75–67.50)
**Sex**	
- Female	21 (65.6)
- Male	11 (34.4)
**Type of tumor**	
**Leiomyosarcoma**	32 (100)
- uterine LMS	5 (15.6)
- retroperitoneal LMS	5 (15.6)
- pelvic LMS	5 (15.6)
- inferior extremities LMS	3 (9.4)
- other sites	6 (18.8)
- not specified LMS	8 (25)
**Grading**	
- 1	1 (2.9)
- 2	6 (17.9)
- 3	16 (47.1)
- NA	11 (32.4)
**Surgery**	
- Yes	28 (87.5)
- No	4 (12.5)
**Dacarbazine (line)**	
- Adjuvant	1 (3.1)
- First line	17 (53.1)
- Second or further	7 (21.9)
- Neoadjuvant	5 (15.6)
- NA	2 (6.2)
**Dacarbazine (method of administration)**	
- Combined	23 (71.9)
- Combined and monotherapy	3 (9.4)
- Monotherapy	5 (15.6)
- NA	1 (3.1)
**Radiotherapy**	
- Yes	17 (53.1)
- No	15 (46.9)
**Best response**	
- PR-SD	25 (78.1)
- PD	6 (18.8)
- NA	1 (3.1)

Abbreviations: IQR: interquartile range; NA: not available; PR-SD: partial response-stable disease; PD: progressive disease.

**Table 2:** Demographic and clinical characteristics stratified according to the presence of methylation.

Characteristics	Methylated, n (%)		*p*-Value
	No	Yes	
**Age (median [IQR])**	56.50 (51.00–65.25)	68.50 (48.75–73.00)	0.220
**Sex**			0.465
- Female	12 (60.0)	9 (75.0)	
- Male	8 (40.0)	3 (25.0)	
**Type of tumor**			
Leiomyosarcoma	20 (100)	12 (100)	
**Grading**			0.792
- 1	1 (1.7)	0	
- 2	4 (8.0)	2 (6.3)	
- 3	11 (30)	5 (24.0)	
- NA	11 (30.0)		
**Surgery**			0.377
- Yes	18 (90.0)	10 (83.3)	
- No	2 (10.0)	2 (16.7)	
**Dacarbazine (line)**			0.568
- Adjuvant	0 (0.0)	1 (8.3)	
- First line	10 (50.0)	7 (58.3)	
- Second or further	4 (20.0)	3 (25.0)	
- Neoadjuvant	4 (20.0)	1 (8.3)	
- NA	2 (10.0)	0 (0.0)	
**Dacarbazine (method of administration)**			0.328
- Combined	14 (70.0)	9 (75.0)	
- Combined and monotherapy	3 (15.0)	0 (0.0)	
- Monotherapy	2 (10.0)	3 (25.0)	
- NA	1 (5.0)	0 (0.0)	
**Radiotherapy**			1
- Yes	11 (55.0)	6 (50.0)	
- No	9 (45.0)	6 (50.0)	
**Best response**			0.174
- PR-SD	17 (85.0)	8 (66.7)	
- PD	2 (10.0)	4 (33.3)	
- NA	1 (5.0)	0 (0.0)	

Abbreviations: IQR: interquartile range; NA: not available; PR-SD: partial response-stable disease; PD: progressive disease.

**Table 3:** Association of MGMT methylation and loss of MGMT protein expression with clinical pathological features.

Characteristics	No. of Samples	Methylated MGMT	Unmethylated MGMT	*p* Value
**Overall tumors**				0.035
Expression	13	3	10	
No expression	7	5	2	
NA	0			
**Grading**				1.00
**Grading 2**				
Expression	3	0	2	
No expression	1	1	0	
**Grading 3**				
Expression	10	2	8	
No expression	3	3	0	
**Type of LMS tumor**				
**Abdominal**				
Expression	2	1	1	
**Gluteus**				0.083
No expression	1	1	0	
Expression	2	0	2	
**Inferior Extremities**				0.025
No expression	1	1	0	
Expression	4	0	4	
**Retroperitoneal**				
No expression	1	1	0	
**Uterine**				0.136
No expression	3	2	1	
Expression	2	0	2	
**Other**				0.505
No expression	1	0	1	
Expression	2	1	3	

## Data Availability

Raw data are available on https://zenodo.org/record/7753644#.ZbiUQnbMJPY (accessed on 20 March 2023).
